# If the antibody fails – a mass Western approach

**DOI:** 10.1111/j.1365-313X.2008.03554.x

**Published:** 2008-07-09

**Authors:** Ute Lehmann, Stefanie Wienkoop, Hendrik Tschoep, Wolfram Weckwerth

**Affiliations:** 1 Max Planck Institute of Molecular Plant PhysiologyAm Mühlenberg 1, 14424 Potsdam, Germany; 2 University of Potsdam, Institute of Biochemistry and BiologyGermany; 3 University of Vienna, Department of Melecular Systems BiologyAustria

**Keywords:** proteomics, mass spectrometry, systems biology, plant systems biology, isozymes, stable isotope dilution

## Abstract

Sucrose-phosphate synthase (SPS) has attracted the interest of plant scientists for decades. It is the key enzyme in sucrose metabolism and is under investigation in various plant species, e.g. spinach, tobacco, poplar, resurrection plants, maize, rice, kiwi and *Arabidopsis thaliana.* In *A. thaliana*, there are four distinct SPS isoforms. Their expression is thought to depend on environmental conditions and plant tissue. However, these data were derived from mRNA expression levels only. No data on SPS protein identification from crude extracts have been available until now. An antibody approach failed to distinguish the four isoforms. Therefore, we developed a method for SPS quantification and isoform-specific identification in *A. thaliana* complex protein samples. Samples were separated on SDS-PAGE, digested and directly applied to liquid chromatography/triple-stage quadrupole mass spectrometry (LC/TSQ-MS). In this approach, known as mass Western, samples were analysed in multi-reaction monitoring (MRM) mode, so that all four SPS isoforms could be measured in one experiment. In addition to the relative quantification, stable isotope-labelled internal peptide standards allowed absolute quantification of SPS proteins. Protein extracts from various plant tissues, samples harvested during the day or the night, and cold-stressed plants were analysed. The stress-specific SPS5a isoform showed increased concentrations in cold-stressed leaf material.

## Introduction

Sucrose synthesis takes place in the cytosol of cells of photosynthetic (Lunn and Furbank, 1997) and non-photosynthetic tissues (e.g. germinating seeds; Weber *et al.*, 1997). It is regulated by fructose-1,6-bisphosphatase (FBPase) (EC 3.1.3.1) (Zimmermann *et al.*, 1978) and sucrose-phosphate synthase (SPS) (EC 2.4.1.14) (Harbron *et al.*, 1981; Pollock, 1976). SPS is a glycosyl transferase. It transfers a glycosyl residue from UDP-glucose (UDP-G) to fructose-6-phosphate (F6P), forming sucrose-6-phosphate (S6P) and UDP. S6P is then further converted to sucrose by sucrose-6-phosphate phosphatase (SPP) (Harbron *et al.*, 1981).

In most plants, sucrose is the main transport metabolite of fixed carbons. It is the main storage reserve in developing sink tissues such as tomato fruits and sugar beet roots. Sucrose is also known to be a signalling molecule in higher plants (Jang *et al.*, 1997; Lalonde *et al.*, 1999; Smeekens, 1998). It modulates the expression of genes that encode enzymes involved in central carbon and nitrogen metabolism (Ciereszko *et al.*, 2001; Stitt *et al.*, 2002), storage proteins (Zourelidou *et al.*, 2002) and enzymes involved in the cell cycle and differentiation (Blazquez *et al.*, 1998; Gaudin *et al.*, 2000; Ohto *et al.*, 2001). Sucrose is also involved in the control of developmental processes such as flowering (King and Ben-Tal, 2001; Ohto *et al.*, 2001), seed development (Iraqi and Tremblay, 2001) and accumulation of storage metabolites (Lozeman *et al.*, 2001; Rook *et al.*, 2001). At low temperature (Malone *et al.*, 2006) and during drought (Yang *et al.*, 2001), sucrose is accumulated as a protective agent and as an energy reserve to reactivate metabolism after conditions have improved.

SPS is a low-abundance protein. In *Arabidopsis thaliana*, there are four SPS isoforms (SPS1, At1g04920; SPS4, At4g10120; SPS5a, At5g11110; SPS5b, At5g20280). They can hardly be identified from crude extracts of greenhouse-grown *A. thaliana* plants using a typical LC/MS shotgun approach as their ion signals are suppressed by more abundant proteins. Immunodetection is an alternative method and SPS antibodies are available. However, even if signals are detected, distinction between the various SPS isoforms is not possible. The same is true for SPS activity assays. There are very sensitive methods that can measure SPS activity in crude Arabidopsis extracts (Gibon *et al.*, 2002), but no conclusions regarding protein amounts or SPS isoforms can be drawn. A selective and more specific tool is therefore needed that allows isoform-specific identification of low-abundance proteins. The mass Western is such a tool.

**Figure 1:**
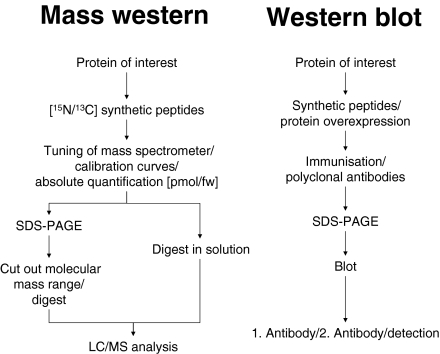
Work flow of mass Western and Western blot/immunodetection methods.

The principle of the mass Western approach presented here is schematically shown in Figure 1. We recently developed a mass spectral reference library for plant proteomics (Hummel *et al.*, 2007), which gives details of tryptic peptides and the mass spectra of the protein of interest for design of synthetic internal stable isotope-labelled peptide standards. These synthetic peptides can be used to optimize the selectivity and sensitivity of a mass spectrometer (Gerber *et al.*, 2003; Glinski and Weckwerth, 2005; Wienkoop and Weckwerth, 2006). Prior to analysis, the proteins of interest are separated on SDS-PAGE, and the specific molecular region is cut out and digested followed by multi-reaction monitoring (MRM) peptide LC/MS analysis. The use of MRM approaches for the accurate quantification of metabolites and proteins in various samples has been discussed by Mayya and Han (2006). Using the method presented here, discrimination between all four *A. thaliana* SPS isoforms was possible, and the protein amounts (in pmol) of each isoform could be determined. The results were verified by SPS activity measurements. All four SPS isoforms were expressed in leaves and flowers. SPS1 (At1g04920) was found to be the most abundant isoform in flowers. SPS5a (At5g11110) levels were increased in cold-stressed leaves compared to samples taken at 20°C.

## Results

### Validation of the method using recombinant SPS

**Figure 2:**
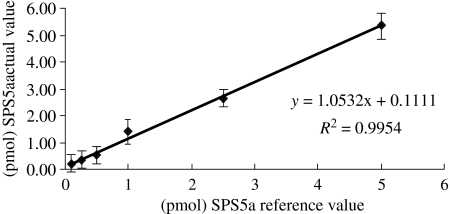
Calibration curve of recombinant SPS5a protein (120 kDa) measured by nanoflow LC-TSQ/MS in MRM mode. Various amounts of transgenic SPS5a were applied to SDS-PAGE (reference values). Bands at 120 kDa were cut out, digested and measured by nanoflow LC/TSQ-MS using labelled internal standards.

The principle of the strategy is shown in Figure 1. The mass Western approach follows a typical Western blotting procedure, but avoids the use of antibodies and is more selective and sensitive. Primary experiments were performed with recombinant SPS. Increasing amounts of purified recombinant SPS5a and a control without SPS were loaded on an SDS gel. The amounts were calculated to range between 100 fmol and 10 pmol. Four replicates per concentration were measured. Gel bands of the corresponding molecular size of SPS were cut out and digested with trypsin. Samples were measured by nanoflow liquid chromatography/triple-stage quadrupole mass spectrometry (LC/TSQ-MS). SPS5a was identified in the samples, and a calibration curve was obtained for quantification (Figure 2). The coefficient of variation for technical replicates is in the range of 15–20%. The limit of detection was in the lower femtomolar range (<10 fmol). According to these limits, the amount of fresh weight has to be adjusted to guarantee SPS protein amounts above the detection limit. Similar results were obtained for more complex samples in which recombinant SPS was spiked into*A. thaliana* crude extracts (data not shown).

To verify the reliability of quantitative and qualitative *A. thaliana* SPS measurements, three distinct recombinant Arabidopsis SPS isoforms SPS1 (At1g04920), SPS5a (At5g11110) and SPS5b (At5g20280) were mixed and prepared for LC/MS measurements. Samples were either digested in solution or applied to SDS-PAGE with subsequent in-gel digestion. Four replicates per condition were prepared. Extracted peptides were measured by LC/TSQ-MS. All three isoforms could be distinguished, and the calculated levels reflected the loaded ranges (data not shown).

### SPS quantification and activity measurements in A. thaliana protein extracts

The method was then tested for *Arabidopsis thaliana* crude protein extracts. Proteins extracted from *A. thaliana* cold-stressed leaves and flowers were enriched by fractionated ammonium sulfate precipitation (30, 40 and 50% ammonium sulfate). Precipitates were taken up in HEPES buffer and desalted. Extracts were used for SPS activity measurements and for SDS-PAGE. For LC/TSQ-MS analysis, gel pieces at 120 kDa were cut out and digested. Values were calculated from four independent samples per condition and two or three standard peptides per isoform.

**Figure 3:**
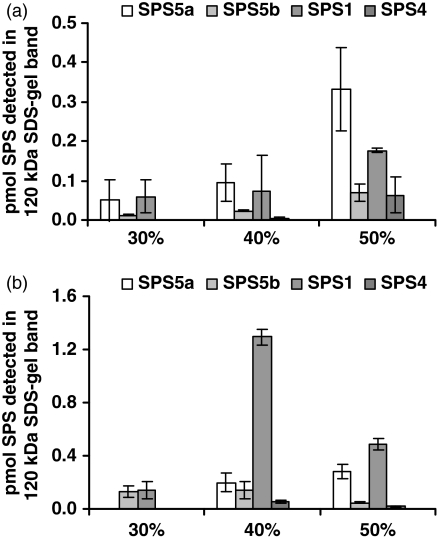
SPS quantification in various *A. thaliana* tissues. (a) SPS quantification in ammonium sulfate precipitates of proteins extracted from cold-stressed leaves. Ammonium sulfate precipitates (30, 40 and 50%) were taken up in native buffer and aliquots applied to SDS-PAGE. The amount of each SPS isoform (in pmol) detected in the 120 kDa gel band is shown. (b) SPS quantification in ammonium sulfate precipitates of extracted flower proteins. Ammonium sulfate precipitates (30, 40 and 50%) were taken up in native buffer and aliquots applied to SDS-PAGE. The amount of each SPS isoform (in pmol) detected in the 120 kDa band is shown.

For both cold-stressed leaf and flower samples, the biological replicates showed high standard deviations (see Figure 3a,b). However, in these protein fractions extracted from *A. thaliana*, clear trends indicating that SPS5a is the dominant isoform in cold-stressed leaves and SPS1 is the dominant isoform in flowers were observed (Figure 3a,b).

**Figure 4:**
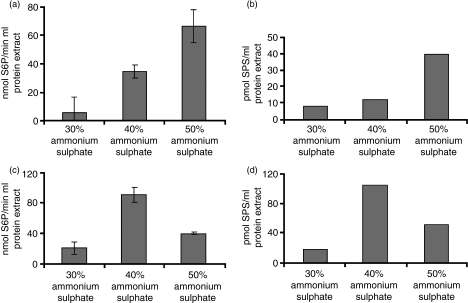
SPS activity measured in ammonium sulfate precipitates of cold-stressed *A. thaliana* leaves (a) and flowers (c) in comparison with the amount of SPS protein detected within the same samples (b and d) by mass Western analysis. SPS amounts (in pmol) are given as the sum of all four SPS isoforms.

To confirm the distribution of SPS isoforms in various ammonium sulfate precipitates, we measured enzymatic SPS activity as maximum activity (*V*_max_) in the same fractions, i.e. 30, 40 and 50% ammonium sulfate precipitates of flower and cold-stressed leaf proteins. Simple measurement of enzymatic SPS activity cannot distinguish between the various isoforms because *V*_max_ reflects the sum of all active SPS enzymes. However, by combining the mass Western with enzymatic activity measurements, we are able to assign the contribution of each SPS isoform to the total activity *V*_max_ (see Figure 3a,b versus Figure 4a,c). The strongest contribution of enzymatic activity is shown by SPS5a in cold-stressed leaf tissue, whereas *V*_max_ correlates more closely with SPS1 in flower tissue. The total enzymatic activity correlates well with the total amount of all SPS isoforms in the corresponding samples (see Figure 4).

### Rapid protein extraction by SDS sample buffer combined with SPS quantification

**Figure 5:**
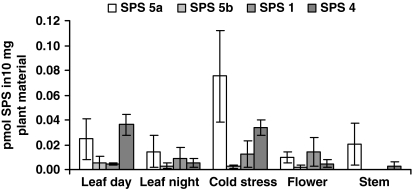
SPS quantification in various *A. thaliana* plant tissues using rapid SDS protein extraction, SDS-PAGE and nanoflow LC-TSQ/MS. The amounts of SPS (in pmol) detected in the 120 kDa gel band(10 mg plant material) as mean value of four technical replicates are shown.

For normal quantification approaches, mild protein extraction is not necessary. Therefore, 10 mg plant material were extracted in 20 μl SDS sample-buffer by boiling the sample for 5 min to determine SPS in different aerial plant parts. Extracts were directly loaded on an SDS gel. Gel pieces at 120 kDa were cut out and prepared for nanoflow LC/TSQ-MS analysis. Leaves harvested during the day or during the night, cold-stressed leaves, flowers and stems were analysed for SPS content. Values were calculated from four independent samples per condition, and two or three peptides per isoform were used as internal standards. The results are shown in Figure 5.

Measured values ranged from 0 to 60 fmol, so were below the optimal quantification range. However, each SPS isoform was identified in the various samples, except for SPS1, which was not found in the stem. As for ammonium sulfate precipitations, SPS5a was found to be the dominant isoform in cold-stressed leaves with increased values compared to unstressed samples. There is a trend towards SPS1 being the most abundant isoform in flowers, in agreement with the results using ammonium sulfate precipitation (see Figure 3b). SPS4 levels decreased from day to night by about sevenfold, as calculated from the mean values, with a coefficient of variation between 25 and 50%. No changes in SPS4 levels were detected in cold-stressed leaves (coefficient of variation between 25 and 16%). SPS5b levels were close to the limit of detection in all samples, with very high standard deviations.

## Discussion

In this paper, we describe a mass spectrometric approach that allows us to distinguish between four SPS isoforms that share 46% (SPS4/SPS1) to 66% (SPS5a/SPSb) sequence homology. Through the use of stable isotope-labelled internal peptide standards, quantification of the distinct isoforms is possible. The work flow for the mass Western approach is shown in Figure 1 and compared with the Western blot procedure. For stable isotope dilution experiments, tryptic peptides from the protein of interest were chosen, chemically synthesized using stable isotope-labelled amino acid substrates, and used as internal standards for identification and quantification. It is important to be aware of the problem of protein phosphorylation. Putative phosphopeptides can hamper the quantification process if chosen as internal standards.

In contrast to the Western blot, the mass Western allows parallel measurement of various proteins of interest in one MS experiment (MRM), whereas for a single blot just one antibody can be used. Another advantage of the mass Western lies in its specificity. It is difficult to raise specific antibodies against very homologous proteins. In such cases, antibodies show strong cross-reactivity. No antibodies are available that allow discrimination of the four Arabidopsis SPS isoforms. The mass Western is the only method that can distinguish between SPS1, SPS4, SPS5a and SPS5b. Our experiments showed that, even at extremely low concentrations (about 10 fmol SPS, approximately 1.2 ng), identification of a distinct isoform is possible.

In addition to identification, absolute protein quantification is possible with the mass Western approach. From our measurements, we estimated the overall content of SPS in leaves of 4- to 5-week-old Arabidopsis plants (in the middle of the light period) to be about 1.65 μg. This is 0.02% of extracted plant proteins (calculated for an average of 2 g leaf material per plant).

Due to the low SPS concentrations within the samples, standard deviations were very high for most of the sample. However, the data indicate some biological trends concerning the concentration of the distinct SPS isoforms. To date, not much is known about the distribution of the four SPS isoforms within *A. thaliana*. It is thought that the distinct SPS isoforms are responsive to different regulatory pathways, and that each isoform is regulated independently (Lunn and MacRae, 2003). The four *A. thaliana* SPS genes and all other known plant SPS sequences can be divided into three families – A, B and C (Lunn and MacRae, 2003). SPS5a and 5b belong to the A family, SPS1 to the B family and SPS4 to the C family. In rice, the B-family gene is expressed in leaves, pollen and germinating seedlings (Chavez-Barcenas *et al.*, 2000). Comparative studies of mRNA expression data led to the conclusion that B-family members are not expressed in roots (Langenkamper *et al.*, 2002). In our studies, SPS1 protein was found to be expressed in leaves and flowers and appeared to be the dominant isoform in flowers. SPS1 was the only isoform that was not detected in stems. The expression of SPS4 has been reported to be under circadian regulation (Harmer *et al.*, 2000). We detected lower SPS4 protein levels during the night compared to those during the day. This is in contrast to observations on mRNA levels. SPS4 mRNA levels increase during the night (Chen *et al.*, 2005; Harmer *et al.*, 2000). An explanation might be that the protein level follows a time shift in the circadian rhythm, as observed for other proteins (Gibon *et al.*, 2004, 2006).

Our studies also suggest that SPS4 and SPS5a are the dominant isoforms in leaves and stems with respect to protein levels. SPS5b levels are comparably low. However, the possibility cannot be excluded that this isoform degrades easily and was not detected in the 120 kDa area of the SDS gel for that reason. It is also possible that the extraction methods used did not pick up SPS5b. However, low amounts of SPS5b protein were detected in flowers, stems and leaves.

For cold-stressed spinach leaves, it is reported that sucrose levels increase after the first day of exposure to 5°C. This sugar accumulation is attributed to four to five times higher levels of SPS enzyme compared to non-stressed plants (Guy *et al.*, 1992). In *A. thaliana* cold-stressed leaves, we found a sevenfold increase in SPS5a levels compared to the unstressed control. These data provide evidence that SPS5a is the stress isoform responsible for cold acclimatization.

To summarize, the method described here provides a tool to distinguish and quantify SPS isoforms. The data obtained complement mRNA expression data and may be completed with information on SPS activity and metabolites. This will allow us to design a complete model of SPS action and to obtain further insight into the complex network of sucrose metabolism. In future we will extend this strategy to whole pathways to study dynamics of metabolic enzymes and to integrate accurate protein concentrations into metabolic network modelling approaches.

## Experimental procedures

### Plant material

Arabidopsis plants (*Arabidopsis thaliana* wild-type Columbia, Col-0) were grown in the greenhouse under controlled conditions (20°C, 55% relative humidity, irradiance 150 μmol m^−2^ s^−1^). Two-week-old seedlings were transferred to GS90 soil (Fritz Kausek GmbH, Germany) and grown under long-day conditions (16 h light, 8 h dark) for 4–5 weeks.

For cold stress, 4- to 5-week-old plants (developmental stage 4–5, Boyes *et al.*, 2001) were transferred to a Percival (CLF plant climatics, http://www.plantclimatics.de/services.htm) at 4°C (irradiance 130 μE) in the middle of the day (after 8 h light). Leaves were harvested 3 days later in the cold.

For flower samples, plants remained in the greenhouse. Flowers were harvested up to flower stage 15 (Smyth *et al.*, 1990). Complete open and closed buds and up to 5 mm long parts of the stem were collected.

### Recombinant SPS

Recombinant *A. thaliana* SPS isoforms (SPS1, SPS4, SPS5a) were expressed in *Escherichia coli* BL21 Codon Plus (DE3) RIL (ARG, ILE, LEU) (Stratagene, http://www.stratagene.com/). Proteins were expressed with a six-histidine tag, and purified from *E. coli* cell lysates by metal-affinity purification using BD Talon metal affinity resin (BD Biosciences, http://www.bdbiosciences.com/index.shtml) in a batch purification procedure. The purification was performed according to the manufacturer's instructions.

### Protein extraction and ammonium sulfate precipitation

Plant material was ground in a mortar and 2.5 g were extracted with 5 ml extraction buffer [50 mm Tris/HCl, pH 8, 20% v/v glycerol, 10 mm DTT, 1 mm EDTA, 20 mm NaF, 0.3 μm microcystin, 1 mm benzamidin, 0.5% v/v proteinase inhibitor cocktail (Roche, http://www.roche.com/home.html), 4 mm leupeptin]. The suspension was kept on ice for 30 min and shaken every 5 min. Crude extracts were centrifuged at 4000 ***g*** for 15 min at 4°C. The supernatant was transferred into a 50 ml tube and subjected to ammonium sulfate precipitation. An appropriate volume of chilled saturated ammonium sulfate solution was added to the extract and the mixture was kept on ice for 1 h. After incubation, it was centrifuged for 45 min at 4000 ***g*** and 4°C. For the second precipitation step, ammonium sulfate solution was added to the supernatant. The procedure was repeated for each precipitation step. Pellets were taken up in 250 μl HEPES buffer (50 mm, pH 8).

### Rapid protein extraction using SDS sample buffer

Plant material was ground in a mortar and 10 mg were extracted in 20 μl SDS sample buffer (225 mm Tris, pH 6.9, 50% glycerol, 5% SDS, 0.05% bromophenol blue, 250 mm DTT). Samples were incubated for 5 min at 95°C and centrifuged for 15 min at 2000 ***g*** at RT. The supernatant was loaded on an SDS gel.

### SDS-PAGE and Coomassie stain

SDS sample buffer (225 mm Tris, pH 6.9, 50% glycerol, 5% SDS, 0.05% bromophenol blue, 250 mm DTT) was added to protein extracts. Samples were heated to 95°C for 5 min and centrifuged for 5 min at 2000 ***g*** at RT. 20 μl were loaded on a 10% acrylamide mini-gel. Electrophoresis was performed as described previously (Laemmli, 1970). Proteins were stained with Coomassie brilliant blue.

### Protein digest and sample preparation

Protein bands were digested with trypsin (Roche) as described previously (Otto *et al.*, 1996). Samples were incubated for 6 h at 37°C without previous alkylation or reduction. Peptides were extracted with increasing acetonitrile concentrations (5, 50 and 90% acetonitrile in 1% formic acid). The extracted peptides were dried in a speed vac (Thermo Fisher Scientific, http://www.thermo.com) prior to analysis or storage at −20°C.

For protein digest in solution, at least one volume of trypsin buffer (10% acetonitril, 25 mm Ambic (ammoniumbicarbonate), 0.5% CaCl_2_) was added to protein samples. Samples were incubated with trypsin beads (GE Healthcare/Amersham Biosciences, http://www5.amershambiosciences.com/) for 6 h at 37°C. Peptide solutions were applied to C18 solid-phase extraction material (Varian, http://www.thermo.com). Elutes were dried overnight and analysed or stored at −20°C.

### Selectivity and sensitivity tuning on a triple-stage quadrupole mass spectrometer

For quantification of all four *A. thaliana* SPS isoforms in *Arabidopsis thaliana* crude extracts, peptide multiple reaction monitoring (MRM) using a triple-stage quadrupole mass spectrometer (TSQ Quantum, Thermo Fisher Scientific, http://www.thermo.com) was used. For quantification, stable isotope-labelled internal peptide standards (^13^C/^15^N leucine) (Thermo Fisher Scientific) were used. Peptide sequences were derived from SPS5a sequences identified in previous analyses of recombinant SPS5a. For SPS1, SPS4 and SPS5b, homologous and experimentally detected peptide sequences were chosen. Two to three peptides for each isoform were selected. To optimize measurements, single reaction monitoring transitions were recorded for each peptide in advance. Peptide sequences, precursor and product ions, and the corresponding collision energies for collision-induced dissociation are listed in Table 1. All the spectra are stored in ProMEX, a plant proteomics mass spectral reference library (Hummel *et al.*, 2007). The corresponding spectra can be visualized, and unknown tandem mass spectra can be searched through the database for peptide/protein identification purposes.

**Table 1:** Target peptides and parameters for quantification of the four SPS isoforms using LC/MS-based high-resolution multiple reaction monitoring

				Precursor ion (*m*/*z*)	Product ion (*m*/*z*)	Collision energy	Precursor ion (*m*/*z*)	Product ion (*m*/*z*)	Collision energy
									
Peptide number	AGI code	Name	Peptide sequence	Standard peptide			Native peptide		
1	At5g11110	SPS5a	DKLEQLLK	497.3	750	25	493.8	743	25
2	At5g11110	SPS5a	VLDNGLLVDPHDQQAIADALLK	789.1	873.9	35	786.8	873.9	35
3	At5g11110	SPS5a	DINDISLNLK	576.3	923.4	26	572.8	916.4	26
4	At5g20280	SPS5b	DKLEQLLR	511.3	778.3	32	507.8	771.3	32
5	At5g20280	SPS5b	VLDNGLLVDPHDQQSISEALLK	805.6	733.5	40	802.1	733.5	40
6	At4g10120	SPS4	NKFEQLLQQGR	456.4	488.1	20	454.2	488.1	20
7	At4g10120	SPS4	ALNNGLLVDPHDQQAISDALLK	784.7	718.4	40	782.4	718.4	40
8	At1g04920	SPS1	DVQDMSLR	485.7	756.1	24	482.2	749.1	24
9	At1g04920	SPS1	GLVTLGSDALLR	611.3	738.3	30	607.8	731.3	30

For each SPS isoform, two or three peptides were chosen as internal standards. Synthetic standard peptides were labelled with ^13^C/^15^N at leucine residues (bold). Precursor ion masses, product ion masses and collision energies are given for internal standards and native peptides.

### LC/MS settings

Mass spectrometry was performed on a TSQ Quantum Discovery MAX mass spectrometer (Thermo Fisher Scientific) operated in the positive-ion mode. The scan width for all MRMs was 0.7 mass units. The resolution for Q1 was 0.2 mass units; that for Q3 was set to 0.7 mass units. The mass spectrometer was tuned to its optimum sensitivity for each standard peptide as described in detail previously (Glinski and Weckwerth, 2005). Table 1 shows the collision energies used for the recorded transitions. The dwell time per transition was 50 msec.

For identification and quantification of the various SPS isoforms, a one-dimensional nanoflow LC system with pre-column (Agilent, http://www.home.aglient.com) was used. A monolithic column (Merck, http://www.merck.com) of 15 cm length and an internal diameter of 0.1 mm was coupled to the TSQ. Peptides were eluted during a 30 min gradient from 5% MetOH to 100% MetOH in 0.1% FA (formic acid) with a controlled flow rate of 0.7 μl min^−1^. Standard peptide (500 fmol) was added to each sample digest prior to analysis (Wienkoop and Weckwerth, 2006).

The spray voltage was set to 1.8 kV and the temperature of the heated transfer capillary was set to 150°C.

### SPS activity measurements

For the analysis of SPS activity in samples of fractionated ammonium sulfate precipitations, protein pellets were taken up in 500 μl HEPES buffer (50 mm, pH 8) and desalted using NAP columns (GE Healthcare, http://www.gehealthcare.com/worldwide.html). Two microlitres of each extract were used for the assay. The method used was as described previously (Gibon *et al.*, 2002, 2004).
